# Postpartum contraceptive use in Gondar town, Northwest Ethiopia: a community based cross-sectional study

**DOI:** 10.1186/s12905-015-0178-1

**Published:** 2015-02-22

**Authors:** Yeshewas Abera, Zelalem Birhanu Mengesha, Gizachew Assefa Tessema

**Affiliations:** Mahiberehiwot for Social Development Organization, Gondar, Ethiopia; Department of Reproductive Health, Institute of Public Health, University of Gondar, Gondar, Ethiopia

**Keywords:** Contraception, Postpartum period, Ethiopia

## Abstract

**Background:**

Addressing family planning in the postpartum period is crucial for better maternal, neonatal and child survival because it enables women to achieve healthy interval between births. The contraceptive behavior of women in the postpartum period is usually different from other times in a woman’s life cycle due to the additional roles and presence of emotional changes. Therefore, this study is conducted with the aim of assessing the contraceptive behavior of women in the postpartum period.

**Methods:**

A community-based cross-sectional study was conducted in August 2013 among women who gave birth one year before the study period in Gondar town, Northwest Ethiopia. Multistage cluster sampling technique was employed to recruit a total of 703 study participants. For data collection, a structured and pretested questionnaire was used. Descriptive statistics were done to characterize the study population using different variables. Bivariate and multiple logistic regression models were fitted. Odds ratios with 95% confidence intervals were computed to identify factors associated with contraceptive use.

**Results:**

Nearly half (48.4%) of the postpartum women were using different types of contraceptives. The most commonly used method was injectable (68.5%). Resumption of mensus [Adjusted Odds Ratio (AOR) = 8.32 95% Confidence Interval (CI): (5.27, 13.14)], age ≤24 years [AOR = 2.36, 95% CI: (1.19, 4.69), duration of 7–9 months after delivery [AOR = 2.26 95% CI: (1.12, 4.54)], and having antenatal care [AOR = 5.76, 95% CI: (2.18, 15.2)] were the factors positively associated with contraceptive use in the extended postpartum period.

**Conclusion:**

Postpartum contraceptive practice was lower as compared to the Ethiopian demographic and health survey 2011 report for urban areas. Strengthening family planning counseling during antenatal care visit and postnatal care would improve contraceptive use in the postpartum period.

## Background

Maternal health problems remain a major global concern since pregnancy and childbirth are the leading causes of morbidity and mortality among reproductive age women. According to 2013 maternal mortality estimate 292, 982 maternal deaths occurred during 2013 and almost 99% of these deaths happened in the developing countries [1]. Moreover, 90% of the neonatal death registered in these countries [2]. According to the Ethiopian Demographic and Health Survey (EDHS) 2011, the maternal mortality ratio is 676 per 100,000 live births [3].

Evidences have shown that encouraging early antenatal care visits, institutional deliveries, postnatal care, and contraceptive adoption are the key elements in improving safe motherhood. As the first pillar of safe motherhood and an essential component of primary health care, contraceptive plays a key role in reducing maternal and newborn morbidity and mortality by preventing unintended pregnancy and close birth intervals [4].

World Health Organization (WHO) technical committee advices an interval of at least 24 months before couples attempt to become pregnant [2]. A closed birth interval would endanger the lives of the mother, the newborn, and the (previously delivered child). When a mother becomes pregnant shortly after childbirth, she is more likely to develop complications including spontaneous abortion, postpartum bleeding, and anemia. Secondly, the newborn could be born low birth weight and/or preterm. Thirdly, the index child (previously delivered child) might receive inadequate care and support which, thereafter, could lead to vulnerabilities to disease and malnutrition [4,5].

In Ethiopia, nearly half of all non-first pregnancies occur less than 24 months following the preceding birth [6]. Another study done in Northwest Ethiopia also showed the presence of short intervals between births [7]. Hence, introduction of effective contraceptive method during the postpartum period is very crucial. Studies have revealed that the first year following delivery is so complex and different from other times in a woman life cycle due to additional burden to care her infant and series of emotional and physical changes [6,8,9]. These women would also perceive a low risk of pregnancy [10,11].

The Ethiopian Health Sector Development Program (HSDP) IV sets a goal of improving maternal health and increasing family planning coverage. However, the first year after birth is given less emphasis regarding contraceptive utilization [12]. Therefore, this study can help health planners and policy makers to develop effective strategies for the prevention of closely spaced and unintended pregnancies.

## Methods

### Study setup

A community-based cross-sectional study was conducted in August 2013 at Gondar town. The town is located 727 kms Northwest of Addis Ababa, capital of Ethiopia. It is divided in to 12 administrative areas. According to the 2013 population projection estimate, there were 258,178 residents and more than half of them were females. Using the conversion factor of 2.77% to estimate the number of women having less than one years old, the estimated number of postpartum women were 5,734 [13]. There are three hospitals and eight health centers providing maternal and other health services to the population. Postpartum women (from 6 weeks to one year of extended postpartum period) who gave birth one year prior to the study period and not pregnant were included in this study.

### Sample size calculation and sampling procedure

**Figure 1:**
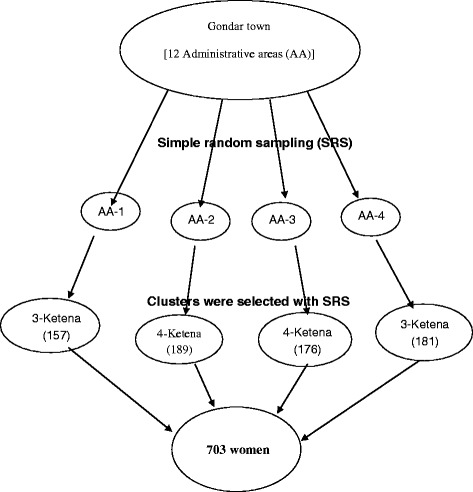
**Schematic representation of the sampling procedure.** *AA- Administrative areas.

The single population proportion formula was used to calculate the sample size considering the following assumptions: Since there is no study in Ethiopia, the proportion of women using contraceptives in the postpartum period was assumed to be 50%, 95% confidence level, 5% margin of error (absolute level of precision). In the recruitment of the study participants, the present study has undertaken multistage cluster sampling technique. In the case of multi-stage sampling approach, design effect should be accounted for the possible presence of inter-cluster variability. With this regard, design effect can be assumed equivalent to the number of stages that had been undergone to reach the final respondents (here there are two stages). However, due to limited resource to conduct the study, we minimized our design effect to 1.5, instead of two. In addition, a non-response rate 10% was considered and finally a sample size of 634 was calculated. In the process of reaching to the individual study participant, a lottery method was employed to select four of the twelve administrative areas. Then, three to four *ketenas* (clusters) in the selected four administrative areas were again randomly selected. Finally, all women in the extended postpartum period were interviewed in each cluster. This made the final number of respondents to be 703 (Figure 1).

### Data collection and analysis

Data were collected using a structured and pretested questionnaire via face-to-face interview at the participant’s home. The questionnaire was first prepared in English and then translated into local language (Amharic), and back to English to ensure consistency. Five midwifery nurses and one supervisor were involved in the data collection process. Local guiders were also participated in recruiting eligible women. Two days training was given to the data collectors and supervisor.

Data were entered using EPI-INFO version 3.5.3 and exported to SPSS version 20 statistical software for further analysis. Descriptive statistics were carried out to characterize the study population using different variables. Both bivariate and multiple logistic regressions were used to identify associated factors. Variables having p value ≤ 0.2 in the bivariate analyses were fitted into a multiple logistic regression model to control the effects of confounding. Crude and adjusted odds ratio with their 95% CI were calculated to determine the strength and presence of association. P value of 0.05 was considered to declare the level of significance.

### Ethical considerations

Ethical clearance was obtained from the Institutional Review Board of the Institute of Public Health, University of Gondar. An official letter of cooperation was written to Gondar town administration. After explaining the purpose of the study, verbal informed consent was obtained from each of the participant. Participants were also informed that participation was on voluntary basis and that they can withdraw at any time if they are not comfortable about the questionnaire. Personal identifiers were not included in the written questionnaires to ensure participants’ confidentiality.

## Results

### Socio-demographic characteristics of the respondents

In this study, 705 women who met the eligibility criteria were included. From these, 703 women responded to the questionnaire making the response rate 99.7%. The mean age of respondents was 27.2 years (SD = 5.7). Two hundred fifty nine (36.8%) were aged between 25–29 years. The majority (86.2%) of the respondent were married. Most (95.6%) were Amhara by ethnicity and 82.1% were Orthodox Christians. Three hundred seventy three (53.1%) were housewives. More than a quarter (28.9%) attended primary school and 39% of the partners attended secondary school (Table 1).

**Table 1:** **Socio-demographic characteristics of the study participants at Gondar town, August 2013 (n = 703)**

**Variable**	**Frequency**	**Percent**
**Age**		
15-19	36	5.1
20-24	198	28.2
25-29	259	36.8
30-34	108	15.4
≥35	102	14.5
**Marital status**		
Married	606	86.2
Single	46	6.5
Separated/widowed/divorced	51	7.3
**Educational attainment**		
No formal education	154	21.9
Primary education	203	28.9
Secondary education	201	28.6
Tertiary education	145	20.6
Husband Educational attainment (n=606)*		
No formal education	42	6.9
Primary education	113	18.6
Secondary education	241	39.8
Tertiary education	210	34.7
**Religion**		
Orthodox Christian	577	82.1
Muslim	107	15.2
Other**	19	2.7
**Ethnicity**		
Amhara	672	95.6
Tigre	30	4.3
Oromia	1	0.1
**Occupational status**		
House wife	373	53.1
Government employee	130	18.5
Merchant	92	13.1
Student	46	6.5
Daily laborer	56	8.0
Other***	6	0.9

*Among those married women **Protestant and Judaism ***job seekers, *Tella (alcohol)* sellers.

### Reproductive health characteristics of participants

The median number of living children was 2.1 per women (IQR = 1.1, 3.0). Two hundred ninety four (41.8%) had only one child. Three hundred and seventy nine (53.9%) did not have desire to have additional children. The median duration of birth interval was 36 months (IQR = 24, 48). Three hundred twenty four (46%) of the respondents did not have intention to have more children. More than half (53.1%) had regular menses. Five hundred two (71.4%) of them had resumed sexual intercourse. One-third (33%) of the respondents were in between 10th-12th month of postpartum period (Table 2).

**Table 2:** **Reproductive health and maternal health service related characteristics of study participants at Gondar town, August 2013 (n = 703)**

**Variable**	**Frequency**	**Percentage**
**Living children**		
1	294	41.8
2-3	322	45.8
≥4	87	12.4
**Fertility Desire**		
Yes	379	53.9
No	324	46.1
Birth interval (in months) (n = 409)*		
<24	90	22.0
24-47	197	48.2
≥48	122	29.8
**Reproductive Intention**		
Want to have space	359	51.1
Want to limit	324	46.1
Undecided	13	1.8
Want to have a child soon	7	1.0
**Menses Return**		
Yes	330	46.9
No	373	53.1
**Postpartum period**		
6th week-3rd month	145	20.6
4th-6th month	146	20.7
7th-9th month	180	25.7
10th-12th month	232	33.0
**Resume sexual intercourse**		
Yes	502	71.4
No	201	28.6
**ANC follow up**		
Yes	631	89.8
No	72	10.2
Number of visit (n = 631)**		
1 visit	3	0.5
2-3 visit	119	18.9
≥4 visit	509	80.7
**Place of delivery**		
Government facility	582	82.8
Private health facility	66	9.7
Home	53	7.5
**Postnatal care**		
Yes	185	26.3
No	518	73.7
FP counseling during PNC***		
Yes	112	60.5
No	74	39.5

*Among those who have previous child. **Among women attending ANC ***Among PNC attendees.

### Maternal health service utilization during last pregnancy

The majority (89.8%) had ANC attendance. From the ANC attendants, 509 (80.7%) had four or more visits. Six hundred forty eight (92.2%) respondents delivered in health facilities. Five hundred fifteen (82.6%) received the service from public health facilities. Among those who had attended ANC, around half (50.2%) were given counseling about family planning. More than a quarter (26.3%) had taken postnatal care service. About a quarter of women (26.3%) were attended postnatal care. One hundred and twelve (60.9%) of the participants received family planning counseling at postnatal care sessions. Two-thirds (66.6%) of the participants had knowledge on Lactational Amenorrhea Method (LAM) (Table 2).

### Contraceptive use in the postpartum period

**Figure 2:**
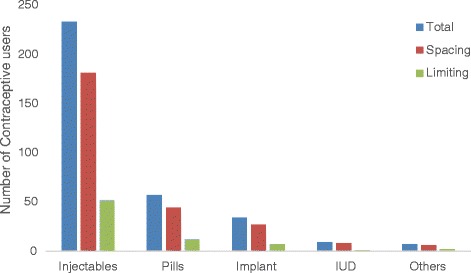
**Number of contraceptive users by purpose among postpartum women in Gondar town, Northwest Ethiopia, August, 2013 (n = 340).**

The prevalence of contraceptive use was found to be 340 (48.4%) [95% CI: (44.5, 52.1)]. Injectable contraceptive 233 (68.5%) and oral contraceptive pills 57 (16.8%) were the most frequently used methods. Among the users, 265 (78%) were using contraception to space their births (Figure 2). Among contraceptive users, 60.5 percent started contraceptive use after the menses resumed. Two hundred forty-eight (72.9%) collected their contraceptives from public health facilities. Two hundred and sixty (76.4%) of the contraceptive users made contraceptive decisions jointly with their partners.

### Reasons for not using contraceptive methods

**Figure 3:**
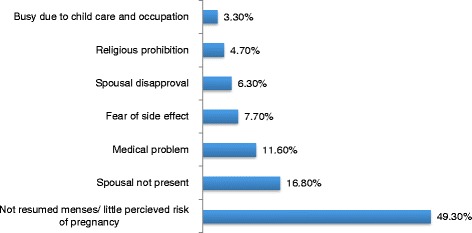
**Reasons not using contraceptive among women in the postpartum period in Gondar town, Northwest Ethiopia, August 2013 (n = 363).**

Less perceived risk for pregnancy (49%) and spousal absence (16.8%) were the main reasons for not using contraceptive methods (Figure 3).

### Factors associated with postpartum contraceptive use

In the multiple logistic regression analysis, age of the women, duration after delivery, menses resumption, and ANC visit showed significant and independent association with postpartum contraceptive use. The odds of using contraceptive were 2.3 times higher among women age <24 years as compared to those who were 35 years or more [AOR = 2.36, 95% CI: 1.19, 4.69]. The odds of using contraceptive were about 2.3 times higher in women 7–9 months after delivery than women between 6 weeks-3 months of postpartum [AOR = 2.26 95% of CI: (1.12, 4.54)]. The odds of using contraceptive in menstruating women were about eight times higher than non-menstruating ones [AOR = 8.32 95% CI: (5.27, 13.14)]. Women who obtained ANC were about five times higher odds to use contraceptive than who did not [AOR = 5.23, 95% CI: (2.04, 13.42)]. Women who were attended postnatal care were about two times higher odds to use contraceptive that those who did not [AOR = 1.63, 95% CI: (1.01, 2.61)] (Table 3).

**Table 3:** **Crude and adjusted odds ratios (OR) and 95% confidence intervals (CI) of factors associated with contraceptive use during postpartum period, Gondar town, August, 2013 (n = 703)**

**Variable**	**Contraceptive use**		**COR (95% CI)**	**AOR (95% CI)**	**P value**
	**Yes**	**No**			
**Age**					*0.00*
≤24	125	109	2.29 (1.41, 3.73)	2.5 (1.04, 6.04)*	
25-34	181	186	1.95 (1.23, 3.08)	1.71 (0.8, 3.65)	
≥35	34	68	1	1	
**Marital status**					0. 62
Not currently married	23	74	1	1	
Currently Married	317	289	2.53 (2.15, 5.79)	2.01 (0.66, 5.01)	
**Educational attainment**					0.58
No formal education	54	100	1	1	
Primary education	104	99	1.96 (1.27, 2.99)	1.27 (0.58, 2.74)	
Secondary & above	182	164	2.06 (1.39, 3.04)	1.98 (0.46, 2.12)	
**Partner educational attainment**					0.68
No formal education	16	26	1		
Primary education	52	61	1.39 (0.67, 2.86)	0.68 (0.26, 1.76)	
Secondary and above	249	202	2.0 (1.05, 3.84)	0.84 (0.32, 2.27)	
**Number of alive children**					0.117
1	154	140	1	1	
2-3	158	164	0.87 (0.63, 1.2)	1.67 (0.97, 2.88)	
≥4	28	59	0.43 (0.26, 0.72)	1.08 (0.41, 2.83)	
**Fertility desire**					0.062
Yes	115	209	0.38 (0.27, 0.51)	0.65 (0.40, 1.06)	
No	225	154	1	1	
**Postpartum period**					0.034
6 wk-3 month	30	115	1	1	
4-6 month	63	83	2.91 (1.73, 4.89)	1.2 (0.67, 2.48)	
7-9 month	114	66	6.62 (4.00, 10.95)	4.8 (2.51, 9.30)*	
10-12 month	133	99	5.15 (3.19, 8.31)	1.9 (1.0, 3.65)	
**Menses return by the time of survey**					*0.000*
No	70	260	1	1	
Yes	270	103	9.73 (6.87, 13.8)	9.2 (5.85, 14.63)*	
**Place of delivery**					0.96
Home	10	43	1	1	
Health institution	330	318	4.46 (2.2, 9.03)	1.02 (0.37, 2.81)	
**ANC care**					*0.001*
No	10	64	1	1	
Yes	330	299	8.88 (4.20, 18.83)	6.61 (2.57, 17.00)*	
**Postnatal care**					*0.042*
No	233	285	1	1	
Yes	107	78	1.68 (1.19, 2.36)	1.63 (1.01, 2.61)	
**LAM knowledge**					0.16
No	137	182	1	1	
Yes	203	181	1.49 (1.11, 2.01)	0.72 (0.45, 1.13)	

*p value<0.05.

## Discussion

Women in the postpartum period have a critical window of opportunity to receive family planning service especially in urban areas because of their better access to health services including ANC, delivery, postnatal care, and immunization [10,14].

This study revealed that nearly half (48.4%) of the participants were using one form of contraceptives. This finding is slightly lower as compared to the 2011 EDHS report for urban women in Ethiopia (52.5%) [3] even though these populations are somehow different from the population in the present study. However, this finding is consistent with studies done in Kenya and Zambia (46%), Mexico (47%), and Rwanda (50.4%) [15-17]. Injectable (68.5%) and pills (16%) were the commonly used methods. Moreover, long acting methods accounted for 12.9% of the users. This would be attributed to client’s preferences for a specific method [17]. These predominant methods have been observed in different studies [3,11,18].

The present study revealed a significant difference in contraceptive use among the different age groups. The odds of using contraceptive were higher among women aged < 24 years than who were 35 years or more. This could be explained by the fact that young women are more sexually active than older women do. A study done in sub-Saharan countries supported this finding [9].

Women whose menses resumed had higher odds to use contraceptive than ammenorrhic women. This might be explained by the fact that ammenhorric women would underestimate the risk of pregnancy by assuming that amenorrhea could guarantee protection against pregnancy regardless of the time of postpartum period. With this regard, in the current study about half (49.3%) of the participants mentioned being ammenhoric as a reason for not using contraceptive. Similar finding was reported from a study done in Kenya [19].

Duration of the postpartum period showed a significant association with contraceptive use. Those women between 7–9 months of postpartum period had higher odds to use contraceptive when compared to women in the 6 weeks −3 months postpartum period. Contrary to this finding, the first three months of postpartum period was reported to be a predictor of contraceptive use [20]. However, consistent results were reported from studies done in Kenya [19] and Bangladesh [21]. This finding could be justified by the fact that most women had resumed menses after 6 months. The other possible reason could be that majority of women were abstainers in the first three months of postpartum period.

ANC utilization was the other important variable affecting contraceptive use. The possible explanation is women who attend antenatal care are more likely to get information towards contraceptive use. This is consistent with a prospective study done in Kenya and Zambia [22]. Studies in Mexico, India and United State of America have shown that FP counseling during prenatal care would motivate women to practice contraceptives [16,20,23]. Those women who were attended postnatal care had higher odds of using contraceptive in postpartum period. This is explained due to that postnatal visit may give the opportunity for contraceptive counseling and adoption in the postpartum period.

This study has some limitations. It mainly focuses on individual level factors. Factors related to the health system and the service providers did not included in the current study. The sociocultural factors and related misconception on family planning did not assessed in this study. Though a sample size of 703 is perceived to be adequate in the present study, due to limited resource to conduct the study, we accounted a design effect of 1.5 in calculating the required sample size.

## Conclusions

The contraceptive use among women in the postpartum period is lower than urban women population in Ethiopia. Resumption of menses, age ≤24 years, duration of 7–9 months after delivery, and having antenatal care were factors positively associated with postpartum contraceptive use. Strengthening the integration of family planning with ANC and postnatal services is recommended to improve the utilization of contraceptives in the postpartum period.
